# Combination epidermal growth factor receptor variant III peptide-pulsed dendritic cell vaccine with miR-326 results in enhanced killing on EGFRvIII-positive cells

**DOI:** 10.18632/oncotarget.15445

**Published:** 2017-02-17

**Authors:** Jianlong Li, Feng Wang, Guangzhi Wang, Ying Sun, Jinquan Cai, Xing Liu, Junhe Zhang, Xiaoyan Lu, Yongli Li, Meng Chen, Lingchao Chen, Chuanlu Jiang

**Affiliations:** ^1^ Department of Neurosurgery, The Second Affiliated Hospital of Harbin Medical University, Harbin 150086, China; ^2^ Neuroscience Institute, Heilongjiang Academy of Medical Sciences, Harbin 150086, China; ^3^ Chinese Glioma Cooperative Group (CGCG), Beijing 100050, China; ^4^ Beijing Neurosurgical Institute, Capital Medical University, Beijing 100050, China; ^5^ Department of Neurology, the Second Affiliated Hospital of Harbin Medical University, Harbin 150086, China; ^6^ Department of Neurosurgery, Huashan Hospital, Fudan University, Shanghai 200040, China

**Keywords:** EGFRvIII, dendritic cell vaccine, miR-326, hedgehog signalling pathway, TGF-β1

## Abstract

The mutant Type III variant of epidermal growth factor receptor (EGFRvIII) is present in approximately one-third of glioblastoma (GBM) patients. It is never found in normal tissues; therefore, it represents a candidate target for GBM immunotherapy. PEPvIII, a peptide sequence from EGFRvIII, was designed to represent a target of glioma and is presented by MHC I/II complexes. Dendritic cells (DCs) have great potential to sensitize CD4^+^ T and CD8^+^ T cells to precisely target and eradicate GBM. Here, we show that PEPvIII could be loaded by DCs and presented to T lymphocytes, especially PEPvIII-specific CTLs, to precisely kill U87-EGFRvIII cells. In addition to inhibiting proliferation and inducing the apoptosis of U87-EGFRvIII cells, miR-326 also reduced the expression of TGF-β1 in the tumour environment, resulting in improved efficacy of T cell activation and killing via suppressing the SMO/Gli2 axis, which at least partially reversed the immunosuppressive environment. Furthermore, combining the EGFRvIII-DC vaccine with miR-326 was more effective in killing U87-EGFRvIII cells compared with the administration of either one alone. This finding suggested that a DC-based vaccine combined with miR-326 may induce more powerful anti-tumour immunity against GBM cells that express a relevant antigen, which provides a promising approach for GBM immunotherapy.

## INTRODUCTION

Glioblastoma (GBM) is the most common primary malignant neoplasm that occurs in the brain, accounting for 60–70% of all gliomas [1–3]. The median survival of patients with GBM is approximately 12–15 months, despite aggressive comprehensive treatment [4] that includes maximal safe tumour resection, followed by chemotherapy and radiotherapy. Therefore, a more efficacious treatment, such as immunotherapy or gene therapy, is urgently needed. One of the most promising immunotherapeutic approaches for the treatment of cancer is adoptive immunity with CTLs stimulated by tumour antigens-pulsed dendritic cells [5], the most robust antigen presenting cell in the immune system. DC immunotherapy exploits a patient's own immune system to induce antitumor immune responses [6].

The type III EGF receptor deletion-mutant (EGFRvIII) is one of the most common mutations in glioblastoma [3]. In the mutant allele, the EGFRvIII gene has an in-frame deletion of 801 base pairs, corresponding to exons 2–7 of mRNA, resulting in the deletion of amino acids 6–273 in the extracellular domain and the generation of a glycine at the fusion point. The newly created tumour-specific epitope is situated near the amino terminus of the receptor extracellular domain [3]. EGFRvIII occurs exclusively in neoplasms, making it an ideal target for antitumor immunotherapy [7].

However, glioblastoma is a rich source of immunosuppressive molecules that may interfere with immune recognition and suppress the clinical strategies of active immunotherapy[8]. The tumour environment, which is often immunosuppressive, contributes to tumour progression and ultimately tumour-associated morbidity. The dominant glioblastoma-associated immunosuppressive factor is the cytokine transforming growth factor (TGF)-β, a multifunctional cytokine that not only interferes with multiple steps of afferent and efferent immune responses, but also stimulates migration, invasion and angiogenesis [8]. Accordingly, reducing TGF-β1 expression is predicted to increase the potency of a DC-based vaccine. The sonic hedgehog (Shh) pathway is a regulatory network that is involved in both development and cancer [9]. Glioma-associated oncogene homologue (GLI), the key molecule triggering this pathway, can increase TGF-β1 expression by binding to its promoter [10]. Our previous research established that miR-326 targeted the 3′-UTR of smoothened (SMO), thereby further inhibiting the sonic hedgehog pathway [11, 12]. Das et al. demonstrated that miR-326 targets the TGF-β1 3′-UTR and decreases its expression in A549 cells [13].

In this present study, we sought to characterize how miR-326 can target the 3′-UTR of SMO and subsequently abrogate Gli expression, thereby reducing the expression and extracellular secretion of TGF-β1. MiR-326 down-regulated the external secretion of TGF-β1 via the SMO/Gli2 pathway instead of by targeting the 3′-UTR of TGF-β1 in the glioma cells, and this resulted in a reversal of the glioblastoma-associated immunosuppressive environment. Moreover, combining a DC-based vaccine with miR-326 resulted in better potency than either treatment administered alone. Therefore, this combined approach may serve as a novel cancer immunotherapy strategy for patients with glioma.

## RESULTS

### PEPvIII-DCs sensitized and accelerated the proliferation of autologous cytotoxic T lymphocytes

**Figure 1:**
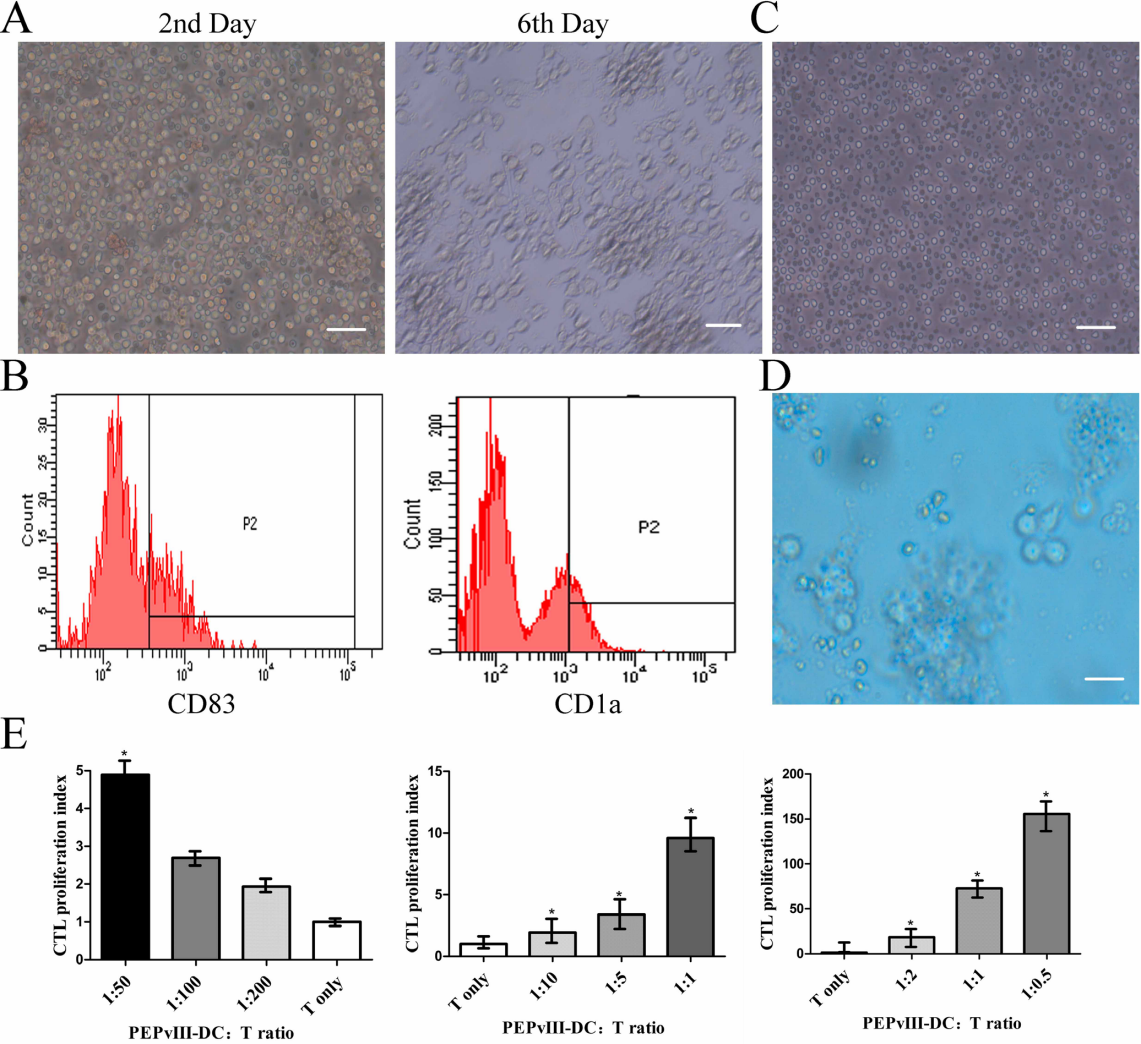
PEPvIII-DCs stimulated T cell proliferation (**A**) Left: PBMCs on the second day. After isolation from peripheral blood, cells were induced with GM-CSF and IL-4 to become MoDC. Right: on Day 6, the morphology of immature DC changed significantly, becoming irregular with dendritic pseudopodium spreading. Bars represent 100 μm. (**B**) Flow cytometry revealed the expression of DC markers, CD83 and CD1a, and confirmed that monocytes had been induced to become MoDC. (**C**) The lymphocytes, in a suspended state, were cultured in RPMI-1640 with 10% FBS. Bars represent 100 μm. (**D**) PEPvIII-DCs and T lymphocytes were co-cultured for 3 days. Bars represent 100 μm. (**E**) Different PEPvIII-DC/T ratios at 1:200, 1:100, 1:50, or 1:10, 1:5, 1:1, and 1:2, 1:1, and 1:0.5 were established, and after co-culturing of DC and T cells for 3 days, a CCK-8 assay was performed to detect the stimulation index by MoDC. These results showed that increasing the amount of co-cultured DCs could promote T cell proliferation; *P < 0.05.

PBMCs were isolated from healthy donators and separated into either adherent or non-adherent cells. Adherent cells were cultured in 250 ng/ml GM-CSF and 250 ng/ml IL-4 for 7 days (Figure 1A). Then, cells were cultured with PEPvIII (40 ug/ml) until day 6 and pulsed with 300 ng/ml LPS on day 7 to obtain mature DCs (Figure 1B) that were able to present antigen to T lymphocytes. The non-adherent cells were mostly lymphocytes (Figure 1C), which were maintained in RPMI-1640 medium with 10% foetal bovine serum. On day 8, PEPvIII-DCs were harvested at a concentration of 1 × 10^6^/ml, with a mDC/T ratio that ranged from 1:200, 1:100, 1:50, or 1:10, 1:5, 1:1, or 1:2, 1:1, 1:0.5. After co-culture for about 3 days (Figure 1D), the cell proliferation capacity was assessed using the CCK-8 assay. It was intriguing that the stimulation index decreased along with the mDC/T ratio from 1:50, 1:100, to 1:200 (Figure 1E). However, these data indicated that the CTLs stimulated by PEPvIII-DCs proliferated the fastest at over 155% when the mDC/T ratio was 1:0.5 compared to unstimulated T cells (Figure 1E).

### PEPvIII-DC-CTLs efficiently killed U87-EGFRvIII cells

**Figure 2:**
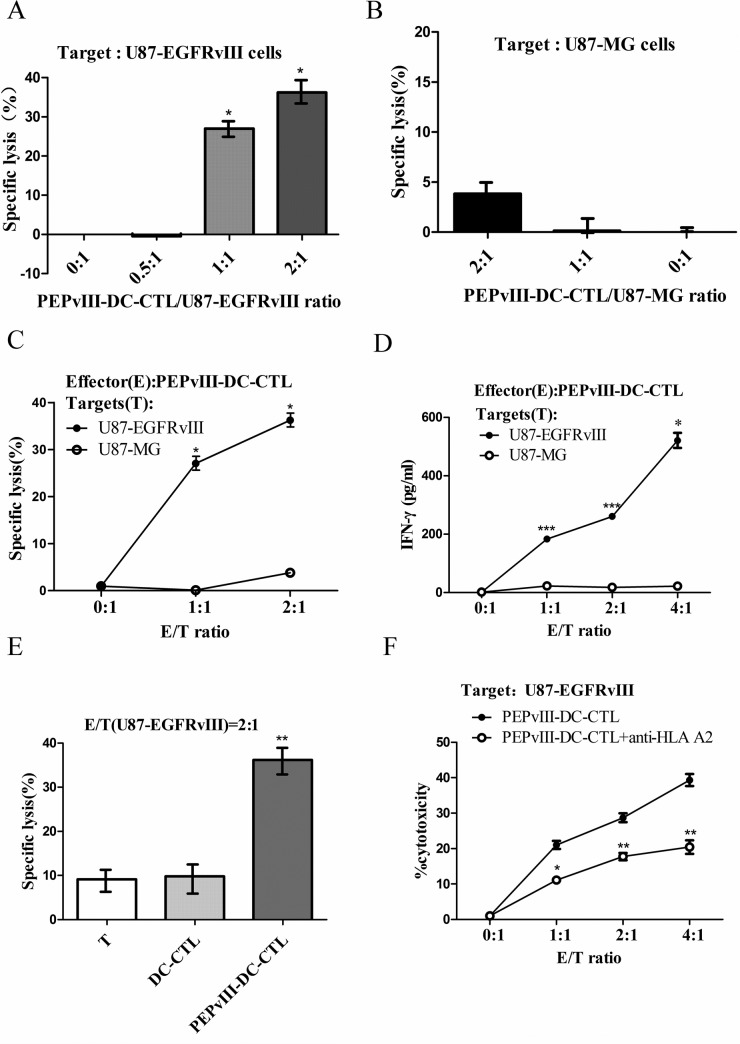
PEPvIII-DC-CTLs efficiently killed U87-EGFRvIII cells Different ratios of effector cells (PEPvIII-DC-CTLs, DC-CTLs or T cells) to target cells (U87-EGFRvIII or U87-MG) were designed to investigate the specific lysis via CCK-8 assay. (**A**) The PEPvIII-DC-CTL to U87-EGFRvIII ratio was 2:1, 1:1, or 0.5:1, and the corresponding cytotoxicity rates were 36.22%, 27.04%, and 0.45%, respectively. The anti-tumour effect increased along with the (effector/target) E/T ratio; *P < 0.05. (**B**) The killing effect of lymphocytes stimulated by PEPvIII-DCs on U87-MG cells. Results showed that the killing rate of U87-MG cells was 3.84% when the E/T ratio was 2:1 or 0.12% at a 1:1 ratio, suggesting that the PEPvIII-DC-CTL had no significant effect on U87-MG cells. (**C**) The induction of EGFRvIII epitope-specific CTLs, which were more efficient in killing EGFRvIII-positive cells. (**D**) PEPvIII-DC-CTLs incubated with U87-EGFRvIII could produce much higher IFN-γ levels than those incubated with U87-MG cells at different E/T ratios (*P < 0.05; ***P < 0.001). (**E**) The killing effect of lymphocytes stimulated or not by DCs that were pulsed with or without PEPvIII on U87-EGFRvIII cells. Compared with DC-CTLs and CTLs, the peptide-DC-CTLs significantly killed the U87-EGFRvIII cells; **P < 0.01. (**F**) Inhibition of the cytotoxic activity of EGFRvIII-specific CTL by monoclonal antibody specific for MHC class I molecules. The target cells were EGFRvIII expressing U87 cells, and the effector cells were activated CTLs stimulated *in vitro* by PEPvIII-peptide pulsed DCs; *P < 0.05, **P < 0.01. These assays were repeated three times; results are shown as mean ± SD.

U87-EGFRvIII and U87 cells were prepared at 1.0 × 10^5^/ml. After co-culture for 20 h and 48 h, cells were tested to determine the killing effects by PEPvIII-DC-CTL using a CCK-8 assay (Figure 2A). Clearly, cell-interactions for 20 h were sufficient for PEPvIII-DC-CTL to target specific glioblastoma cells that expressed the EGFRvIII peptide. Next, we performed CCK-8 assays to investigate the killing effects of PEPvIII-DC-CTLs on U87-MG cells without EGFRvIII. As shown in Figure 2B, the cytotoxicity of U87-MG was 3.84% when the E/T ratio was 2:1 and 0.12% at 1:1. Our preliminary data (Figure 2C), along with our data presented in Figure 2A and 2B, indicated the induction of EGFRvIII epitope-specific T cells, which could specifically lyse EGFRvIII-expressing targets in an E/T ratio-dependent manner. To further characterize this finding, we performed IFN-γ ELISA, because IFN-γ secretion correlated with the cytolytic ability of effector cells [14–19]. As shown in Figure 2D, PEPvIII-DC-CTLs incubated with U87-EGFRvIII produced significantly higher levels of IFN-γ than those CTLs incubated with U87-MG (*P < 0.05; ***P < 0.001). Moreover, consistent with the lysis of target cells, PEPvIII-DC-CTLs co-cultured with U87-EGFRvIII cells secreted IFN-γ in an E/T ratio-dependent manner. In these assays, the nonspecific lysis of irrelevant targets was subtracted from the observed responses to indicate the specific killing.

To study the importance of peptide-pulsed DC in killing tumour cells, different assays containing T cells, DC-T or PEPvIII-DC-CTL were used as effector cells. Our findings indicated that T cells or DC-T cells hardly killed U87-EGFRvIII, reaching approximately 9.64% and 9.14% of killing efficacy, respectively, which was significantly less than that of PEPvIII-DC-CTLs (Figure 2E). Additionally, the IFN-γ suspended in medium was captured using an IFN-γ ELISA Kit. This finding indicated that after mixing with U87-EGFRvIII cells, PEPvIII-DC-CTLs secreted more IFN-γ than DC-CTLs or T lymphocytes despite the E/T ratio (Table 1).

**Table 1:** IFN-γ ELISA of various effectors incubated with the same targets, U87-EGFRvIII cells

IFN-γ ELISA (pg/ml)			
Effector/Target ratio	PEPvIII-DC-CTL to U87-EGFRvIII	DC-CTL to U87-EGFRvIII	T to U87- EGFRvIII
2:1	38.63	0	0
4:1	28.91	15.51	2.31
8:1	43.16	0.33	1.93
*P*-value		< 0.01	0.012

Abbreviations: EGFRvIII: type III variant of epidermal growth factor receptor; IFN-γ: gamma-interferon; ELISA: Enzyme-linked immunosorbent assay; PEPvIII: EGFRvIII peptide; DC: dendritic cell; CTL: cytotoxic T lymphocyte.

Since U87-EGFRvIII cells are derived from U87 glioma cell line and U87 cells are HLA a*0201 positive [20], we further demonstrated that the recognition of the EGFRvIII peptide by PEPvIII-DC-CTLs was HLA-A2 restricted. Blocking assays using a monoclonal anti-HLA-A2 antibody were performed. As shown in Figure 2F, the killing of U87-EGFRvIII cells by EGFRvIII peptide specific CTL was significantly suppressed by the anti-HLA-A2 blocking antibody. It indicates that EGFRvIII peptide is presented on HLA-A2 molecules and the killing of EGFRvIII-expressing glioma cells by the peptide-specific CTLs is HLA-A0201 restricted. Together, these data suggested that the PEPvIII-DC-CTLs specifically targeted tumour cells that expressed EGFRvIII in a HLA-A2 manner and induced cell immunity via IFN-γ.

### TGF-β1 repressed the effector functions of PEPvIII-DC-CTLs

**Figure 3:**
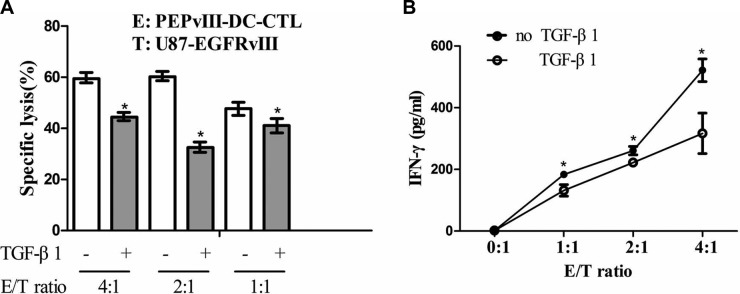
TGF-β1 repressed the effector functions of PEPvIII-DC-CTLs (**A**) The effector-to-target cell ratio ranged from 4:1 to 1:1. After adding recombinant TGF-β1 (75 ng/ml) protein to the co-culture system of PEPvIII-DC-CTLs and U87-EGFRvIII cells, the killing efficacy was partially, but significantly suppressed; *P < 0.05. (**B**) TGF-β1-mediated suppression of IFN-γ release by activated PEPvIII-DC-CTLs. Activated PEPvIII-DC-CTLs were cocultured with U87-EGFRvIII glioma cells in the absence or presence of TGF-β1. Levels of IFN-γ secreted by CTLs were quantified by ELISA. E = PEPvIII-DC-CTL; T = U87-EGFRvIII; *P < 0.05.

As TGF-β signalling in tumour-specific CTLs dampens their function and frequency in tumour [21] and it has been reported that TGF-β-mediated inhibition of CTL function during tumour immunity may occur via several mechanisms [21–27], we investigated whether TGF-β1 could suppress the function of DC-based vaccine in killing tumour cells. After the addition of rhTGF-β1 at a concentration of 75 ng/ml to the co-culture system of PEPvIII-DC-CTLs and U87-EGFRvIII cells, the killing efficacy was significantly suppressed, irrespective of the E/T ratio (Figure 3A). Activated PEPvIII-DC-CTLs were cocultured with U87-EGFRvIII cells in the presence of rhTGF-β1 for 20 h. The addition of TGF-β1 reduced the level of IFN-γ secretion by PEPvIII-DC-CTLs at all E/T ratios tested, as shown in Figure 3B. In conclusion, TGF-β1 suppresses the effector functions of CTLs by reducing cytolytic capacity and IFN-γ release.

### MiR-326 inhibited TGF-β1 expression and secretion by down-regulating the SMO/Gli2 pathway

**Figure 4:**
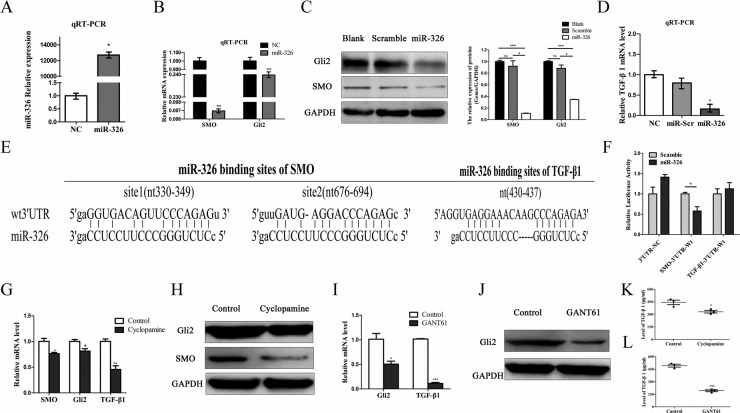
MiR-326 inhibited TGF-β1 expression by the down-regulation of the SMO/Gli2 pathway (**A**) U87-EGFRvIII cells were transfected with miR-326 mimics or miR-Scramble for 48 h. Overexpression of miR-326 was confirmed by qRT-PCR. (**B**) qRT-PCR analysis indicated that the overexpression of miR-326 significantly reduced the SMO and that of its downstream target, Gli2, compared with the miR-Scr-treated group. The data represent the mean ± SE of three replicates. **P < 0.01. (**C**) Left: the upregulation of miR-326 reduced levels of SMO and Gli2 protein expression. Right: quantitative analysis of the grey intensity was determined. The data are representative of three independent experiments. NS: not significant. *P < 0.05; ***P < 0.001. (**D**) qRT-PCR analysis revealed that the TGF-β1 mRNA expression levels of U87-EGFRvIII cells transduced with miR-326 were inhibited when compared with miR-Scr. Expression levels were normalized to those of GAPDH; *P < 0.05. Error bars = SD. (**E**) Diagram of the seed sequence of miR-326 that matched the 3′-UTR of the SMO and TGF-β1 genes. Each of the 3′-UTR constructs was incorporated into reporter constructs. (**F**) Relative Firefly/*Renilla* luminescence (mean ± SD, *n* = 3) mediated by a luciferase plasmid harbouring the wild-type 3′-UTR of SMO or TGF-β1 sequence upon transfection with miR-326. The signal indicates direct interactions between miR-326 and SMO, rather than TGF-β1; *P < 0.05. (**G**) U87-EGFRvIII glioma cells were treated with SMO inhibitor, Cyclopamine (10 μM) for 36 h. SMO, Gli2 and TGF-β1 expression were determined by qRT-PCR, (**H**) Western blot analysis, and (**K**) ELISA. (**I**) U87-EGFRvIII glioma cells were treated with GLI inhibitor, GANT61 (5 μM) for 36 h. Gli2 and TGF-β1 expression were determined by qRT-PCR, (**J**) Western blot analysis, and (**L**) ELISA. GAPDH was used as the loading control. The data represent the mean ± SE of three replicates (*P < 0.05; **P < 0.01; ***P < 0.001).

To investigate the effect of miR-326 in a reversal of the glioblastoma-associated immunosuppressive environment, we first transfected U87-EGFRvIII cells with miR-326 and the efficiency of miR-326 was confirmed by qRT-PCR analysis (Figure 4A). Upregulation of miR-326 significantly decreased the expression of SMO along with its downstream target, the transcriptional factor Gli2 (Figure 4B, 4C). Previous studies revealed that Gli2 increases the expression of TGF-β1, as Gli2 can target at least two sites in the human TGF-β1 promoter [10]. Herein, qRT-PCR analysis confirmed that miR-326 had a negative effect on TGF-β1 expression (Figure 4D). Moreover, we performed TGF-β1 ELISA to measure the quantity of TGF-β1 in the supernatants of U87-EGFRvIII cells transfected with miR-326. Clearly, the amount of TGF-β1 was decreased in the supernatant after miR-326 upregulation when compared with the control group (Table 2).

**Table 2:** TGF-β1 ELISA revealed that miR-326 inhibited TGF-β1 secretion by U87-EGFRvIII cells

TGF-β1 ELISA (pg/ml)			
Number	NC	miR-326	
1	301.66	151.97	
2	326.97	260.74	
3	278.51	167.05	
Average	302.38	193.25	
P-value	0.041		

Abbreviations: TGF-β1: Transforming growth factor-beta 1; ELISA: Enzyme-linked immunosorbent assay; EGFRvIII: type III variant of epidermal growth factor receptor; DC: dendritic cell; CTL: cytotoxic T lymphocyte.

To confirm whether SMO/Gli2 or TGF-β1 was a direct target of miR-326, we first used the miRanda, miRecords and Targetscan algorithms to identify SMO and TGF-β1 as candidate targets for miR-326 (Figure 4E). We constructed the wild luciferase reporter plasmids GV272-TGF-β1-3′-UTR and GV272-SMO-3′-UTR that contained a putative miR-326 binding site of the TGF-β1 and SMO 3′-UTR, respectively, downstream of the luciferase open reading frame. Next, we transiently expressed these constructs in U87-EGFRvIII cells in the presence or absence of miR-326 mimics. Reporter assay revealed that a significant 30–50% reduction in luciferase activity occurred at the GV272-WT-SMO-3′-UTR in the presence of miR-326 mimics, while no significant change in GV272-WT-TGF-β1-3′-UTR was observed (Figure 4F). These data suggest that miR-326 binds to the 3′-UTR of SMO instead of TGF-β1, at least in glioma cells, and impairs SMO and processes downstream of Gli2 mRNA translation. Moreover, we sought to determine whether miR-326 primarily inhibited TGF-β1 through downregulation of SMO/Gli2. A SMO inhibitor, Cyclopamine [4] and a GLI inhibitor, GANT61 [28] was used to block the Hh signalling pathway for 36 h. qRT-PCR, Western blotting and ELISA results indicated that SMO, Gli2 and TGF-β1 expression decreased in cells treated with Cyclopamine, comparing with Control group (Figure 4G, 4H and 4K). Similarly, expression levels of Gli2 and TGF-β1 decreased after being treated with GANT61 (Figure 4I, 4J and 4L). These data suggested that TGF-β1 was regulated by SMO/Gli2 activity. In conclusion, miR-326 reduced TGF-β1 expression by downregulating the SMO/Gli2 pathway.

### EGFRvIII peptide-pulsed DCs and miR-326 synergized to induce more potent immune responses

**Figure 5:**
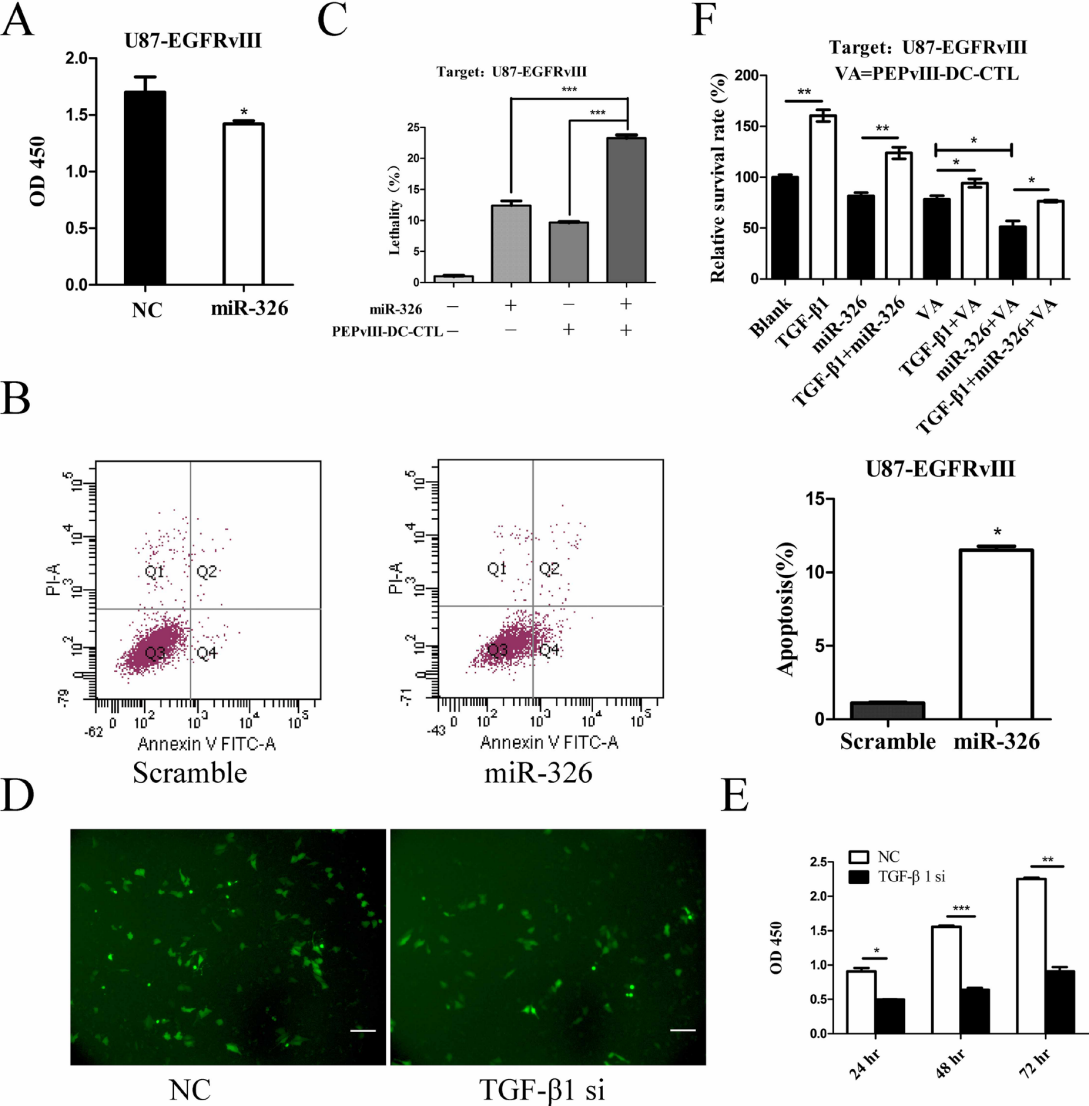
The synergistic effects of EGFRvIII peptide-pulsed DCs and miR-326 on U87-EGFRvIII cells (**A**) A representative cartogram showing glioma cell proliferation inhibited by miR-326 overexpression; *P < 0.05. (**B**) Annexin V-PI assay reveals increased apoptosis in U87-EGFRvIII cells following transfection with miR-326. The early apoptosis rate was quantified; *P < 0.05. (**C**) CCK-8 analysis of U87-EGFRvIII cells revealed changes in cell lethality. U87-EGFRvIII cells were treated with either miR-326 or EGFRvIII peptide-pulsed DCs alone or both together. This analysis revealed that the killing effect of combining these two treatments was significantly greater compared with the control groups in which U87-EGFRvIII cells were either treated with PEPvIII-DC-CTLs or transfected with miR-326; ***P < 0.001. (**D**) Representative images under fluorescence microscope showed the transfection efficiency. Cells transfected with TGF-β1 siRNA or NC plasmid were confirmed by its GFP protein expression. Bars represent 50 μm. (**E**) Cell viability was examined with CCK-8 assay in different time intervals after siRNA treatment. *P < 0.05, **P < 0.01, **P < 0.001. (**F**) U87-EGFRvIII cells were pre-treated with rhTGF-β1 or medium, then followed by miR-326, CTLs or both of them respectively. Cells survival rate was detected using a CCK-8 assay. *P < 0.05, **P < 0.01. The data represent the mean ± SE of three replicates.

To assess the tumour suppressor potential of miR-326, we transfected U87-EGFRvIII cells with miR-326 and then subjected them to proliferation and apoptosis analyses. Overexpression of miR-326 led to reduced proliferation and increased apoptosis compared with the scramble control (Figure 5A, 5B). To investigate whether miR-326 improved the killing effect of single dendritic cell vaccine, we exposed miRNA-transfected target cells to EGFRvIII-DC-CTLs. The concentration of PEPvIII-DC-CTLs and U87-EGFRvIII cells transfected with miR-326 were adjusted to 1.0 × 10^5^/ml and 2.5 × 10^4^/ml, respectively. Then, 24 h later, a CCK-8 assay was performed to assess the rate of target cell killing. DC vaccine treatment of glioma cells led to a slight increase in the lethality rate. Notably, the cytotoxicity of PEPvIII-DC-CTLs was markedly enhanced in the miR-326 miRNA-transfected groups, when compared with DC vaccine alone (Figure 5C).

To demonstrate that miR-326 potentiated the CTLs recognition and killing through a TGF-β1 mediated mechanism, we transfected U87-EGFRvIII cells with a vector expressing siRNA specific for TGF-β1 followed by functional assay. Fluorescence microscope confirmed effective transfection of TGF-β1 siRNA into glioma cells (Figure 5D). As expected, treatment with TGF-β1 siRNA decreased proliferation in U87-EGFRvIII cells (Figure 5E). In addition, when U87-EGFRvIII cells were pre-treated with rhTGF-β1 and then transfected with miR-326 mimics, we observed that TGF-β1 significantly reversed the cells viability (Figure 5F). These results (Figure 4D, Figure 5 and Table 2) suggest that the antitumor activity of miR-326 is mediated by TGF-β1 blockade. Furthermore, to identify that miR-326 treatment and CTL recognition synergized to kill glioma cells through a TGF-β1-mediated mechanism, we pre-incubated U87-EGFRvIII cells with rhTGF-β1 or medium for 12 hours. Then these cells were treated with miR-326, PEPvIII-DC-CTL or both of them. As shown in Figure 5F, TGF-β1 alone increased the viability of tumor cells. The cytotoxicity of miR-326, PEPvIII-DC-CTL or combination of them were significantly rescued by TGF-β1. Taken together, this finding indicates that miR-326 transfection potentiates the EGFRvIII-DC-CTL-induced killing effect in glioma cells by down-regulating TGF-β1.

## DISCUSSION

Glioblastoma, characterized by a high capacity to proliferate and invade, is refractory to traditional surgical tumour resection supplemented with chemotherapy or radiotherapy. Thus, the outcome of GBM patients remains dismal [29]. Recently, many studies have reported potential novel malignant glioma treatments, among which the most promising is immunotherapy [30]. This strategy utilizes the host immune system to specifically recognize damaged or mutated cells and destroy them without off-target effects on the surrounding tissues, unlike radiotherapy or chemotherapy [31].

As a major immune cell type, DCs migrate and distribute in the peripheral tissues where they encounter foreign pathogens or cells. DCs uptake antigens and process and then present them to T cells in lymph nodes (LNs) in the form of peptide-MHC molecules [32]. DCs also provide a second signal, including CD80/CD86 or CD40, which are indispensable for inducing T cell activation and immune responses. Recently, several peptide vaccines have been reported, such as interleukin-13 receptor alpha 2 (IL-13Ra2) [5] and an EphA2 [33] peptide-pulsed DC vaccine.

Known glioma-associated antigens (GAAs) include the IL13Ra2, HER2, gp100, TRP2, EphA2, survivin, WT1, SOX2, SOX11, MAGE-A1, MAGE-A3, AIM2, SART1, and CMV proteins. In addition, EGFRvIII and the IDH1 mutant (R132H) represent truly tumour-specific targets that occur within a subset of tumours [34]. Herein, we focused on EGFRvIII because it is a glioma-specific antigen (GSA), which is only expressed in tumours, and not in normal tissues. Wu et al [35] provided evidence that EGFRvIII induced MHC-restricted CTLs by DCs. In our present study, we also demonstrated that PEPvIII-DCs sensitized and accelerated the proliferation of autologous cytotoxic T cells which could destroy target cells that presented PEPvIII on their surface. In addition, PEPvIII-DC-CTLs were non-toxic towards U87-MG cells, a cell line that does not present the antigenic peptide EGFRvIII. More interestingly, DCs that have not been exposed to antigen become much less potent in killing specific target tumour cells. However, it is notable that not all glioma cells express this antigen. Once GBM recurs, most patients lost EGFRvIII expression as a result of the elimination of a specific population of tumour cells that express EGFRvIII or downregulation the expression of a mutated tyrosine kinase [1]. Multiantigenic vaccines may become an alternative approach to expand the range of vaccine applications. However, care must be taken to avoid autoimmunity. Perhaps, diverse vaccines could be generated to target different GSAs because of the heterogeneity of GBM patients.

In addition, potent self-protection from GBM and the presence of an immunosuppressive tumour environment comprised of Treg cells and marrow-derived stem cells (MDSCs) that produce IL-10 and TGF-β1 reduces the killing effects of DC-based vaccines on tumour cells. To some extent, inhibition of TGF-β1 may also induce autoimmunity because TGF-β1 plays a key role in regulating the homeostasis of the immune system [36]. Therapeutically, it would be useful to reduce the activity of TGF-β1 and then closely monitor the response. Based on the findings of Du [12] and Furler R [10], we further studied how to inhibit the expression of TGF-β1. We found that miR-326 could suppress the expression and external secretion of TGF-β1 in GBM via the SMO/Gli2 signalling pathway, which could at least partially reduce the stress of immunosuppressive environment, and therefore enhance the effect of a DC vaccine. Moreover, crosstalk between miR-326 and TGF-β1 occurred via the HH signalling pathway. For the first time, we showed that miR-326 inflicted pressure on the tumour environment, which expanded the range of regulation by microRNA. Most importantly, we reported for the first time that a combination miR-326 and DC-based vaccine increased the immune response of a DC-vaccine, compared with miR-326 or a DC vaccine alone. This provided a novel approach for glioma immunotherapy treatment.

To date, antigens that are expressed on all glioma cells but not normal tissues have not been found. In the future, more attention should be aimed at identifying this type of antigen to avoid side effects. If so, it could omit the pre-clinical screening and shorten the time to prepare a specific vaccine after glioma is diagnosed, thereby limiting tumour-derived immunosuppression and improving the outcome of glioma patients.

In summary, we tested and verified that PEPvIII-DC-CTLs could specifically kill U87-EGFRvIII cells. Moreover, TGF-β1 secreted by U87-EGFRvIII cells counteracted this process. However, miR-326 inhibited the expression and external secretion of TGF-β1 via the SMO/Gli2 pathway, thereby reversing the immunosuppressive environment. Most importantly, a combination of the two approaches was more beneficial than any one alone. These findings suggest that a DC-based vaccine combination with miR-326 can serve as a novel approach for glioma immunotherapy that may translate into better clinical outcomes.

## MATERIALS AND METHODS

### Cell culture

Human glioma cell line U87-MG were purchased from the Chinese Academy of Sciences Cell Bank. U87-EGFRvIII was a kind gift from Prof. Chunsheng Kang (Tianjin Neurological Institute, Tianjin Medical University General Hospital) [37]. U87-EGFRvIII was used in our study, for it has been engineered to express and present the EGFRvIII antigen. Both U87-EGFRvIII and U87-MG cells were maintained in Dulbecco's modified Eagle's medium (DMEM, Hyclone, USA) supplemented with 10% foetal bovine serum (FBS, Gibco, USA), and additionally 400 μg/ml G418 (Amresco, USA) for U87-EGFRvIII at 37°C in a humidified atmosphere with 5% CO_2_ and 95% air.

### Peptide, rhTGF-β1, Cyclopamine and GANT61

EGFRvIII peptide, PEPvIII (LEEKKGNYVVTDHC) (Genscript, USA), is a 13-amino-acid peptide with an additional terminal cysteine that spans the EGFRvIII mutation. The peptide preparation was greater than 95.0% pure, as assessed by high pressure liquid chromatography. It was dissolved as recommended in ultrapure water to yield a stock solution (1000 μg/ml) and further dissolved in RPMI-1640 with 10% FBS to generate a working solution at a concentration of 40 μg/ml. Recombinant Human TGF-beta 1 (rhTGF-β1, Peprotech, USA) was used at 75 ng/ml. GANT61 was purchased from Selleck Chemical (USA), dissolved in absolute ethyl alcohol (ethanol) to make a stock solution (10 mg/ml). Ethanol concentrations were kept below 0.1% in all cell cultures. Cyclopamine (Sigma-Aldridge, USA) was dissolved in DMSO and was used at a final concentration of 10 μM.

### Generation of mDC and T lymphocytes

Human monocyte-derived dendritic cells (MoDC) were separated from peripheral blood monocyte cells (PBMCs) of healthy HLA A0201 volunteers (Supplementary Figures and samples) using Ficoll-Hypaque centrifugation (TBDscience, China) according to the manufacturer's instructions. Informed consent was obtained from all volunteers involved in this study. The experimental protocols were approved by the Clinical Research Ethics Committee of the 2nd Affiliated Hospital of Harbin Medical University (2013-R-024) and was performed according to the principles of the Helsinki Declaration and Good Clinical Practice. As previously described [14, 35, 38], after isolation from blood, PBMCs were maintained in RPMI-1640 (Hyclone) supplemented with 10% FBS, penicillin (100 U/ml, Beyotime, China) and streptomycin (100 μg/ml, Beyotime) for 3 h to allow for plastic adherence. Then, suspended cells were harvested into another flask for lymphocyte culture, and adherent cells were cultured in recombinant GM-CSF (250 ng/ml, Peprotech) and recombinant IL-4 (250 ng/ml, Peprotech) to induce into MoDC. Every other day, half of the medium was replaced with fresh one. At day 6 of culture, immature DC (iDC) were pulsed with PEPvIII peptide at a concentration of 40 μg/ml; the next day, the pro-inflammatory cytokine LPS (300 ng/ml, 0111:B4, Sigma-Aldrich, USA) was added into the culture medium to induce MoDC to become mature DC (mDC) [39]. On the 8th day, mature dendritic cells (mDC, final product) were collected, allowing them to sensitize lymphocytes [40]. The T cell-enriched nonadherent fraction of PBMCs obtained following the DC plastic adherence step was cultured in RPMI-1640 cell culture medium supplemented with 20 U/ml human IL-2 (Peprotech) and was used for CTL generation [14].

### CTL assay

PEPvIII-pulsed DCs were co-cultured with T lymphocytes for 3 days to obtain antigen-specific killing cells/cytotoxic T lymphocytes (CTLs). For the CTL assay, CCK-8 kit (Dojindo, Japan) and IFN-γ ELISA kit (USCN, USA) were used according to the manufacturer's instructions. Briefly, U87-EGFRvIII cells (targets, T) transfected with or without miR-326 were adjusted to a concentration of 1.0×10^5^/ml, and PEPvIII-DC-CTLs (effectors, E) were adjusted to a concentration of 2.0×10^5^/ml. For each well in a 96-well plate, 200 μl cell suspension of E/T was added, followed by a brief centrifugation. Then, it was kept in an incubator for 70 h, and 20 μl (10%) CCK-8 was added to each well. Subsequently, the OD was read in the IMARK. Specific lysis (%) was calculated according to the following formula: 1-(C-P)/(U-B)×100, where C = co-cultured OD; P = PEPvIII-DC-CTL control OD; U = U87-EGFRvIII OD; B = Blank OD. Additionally, the suspension was harvested for IFN-γ ELISA. For the antibody blocking experiments, PEPvIII-DC-CTLs were incubated with anti-HLA-A2 monoclonal antibody (Proteintech, USA) for one hour before the assay.

### Enzyme-linked immunosorbent assay (ELISA)

Cell suspensions were collected into Eppendorf Tubes and centrifuged at 1000 × g for 20 min, and then the clear supernatant was carefully pipetted into a clean tube using a Pasteur pipette. Samples were aliquoted into pre-labelled plastic, screw-cap vials and stored at −80°C; freeze-thaw cycles were avoided, or the sample was assayed immediately using an indirect ELISA method, as per the manufacturer's instructions. Absorbance at 450 nm was measured using an IMARK microplate reader. Kits used in this test were the IFN-γ ELISA (USCN, USA) and TGF-β1 ELISA (Boster, China) kits.

### Cell transfection

Approximately 1 × 10^5^ U87-EGFRvIII cells during the exponential phase were plated on each well in a 12-well plate (1000 μL/well) to ensure that the cell confluence reached 60–90% at the second day. Then, 2 μl Lipofectamine 2000 transfection reagent (Invitrogen, USA) and 2 μl miR-326 mimics or TGF-β1 siRNA (GenePharma, China) were added into separate tubes with 50 μL DMEM for 5 min; then, they were mixed together for 15–20 min. Next, the mixture was added directly to cells in culture medium in the presence of serum but no antibiotics. At 4 to 6 h after transfection, medium was removed and replaced with fresh media, and the culture was continued for an additional 42–68 h. The human TGF-β1 specific-siRNA sequences GATCCCC*GACTATCGACATGGAGCTG*ttcaagaga*CAG CTCCATGTCGATAGTC*TTTTTGGAAA and TCGATTT CCAAAAA*GACTATCGACATGGAGCTG*tctcttgaa*CAG CTCCATGTCGATAGTC*GGG were cloned into the pGPU6/GFP/Neo vector (GenePharma). Photographs of the transfected cells were taken at 0 h and after 24 h using an Axiovert 200 microscope (Carl Zeiss) before CCK-8 assay. The fluorescence was captured in six different photographs.

### RNA extraction

Total RNA was extracted using TRIzol reagent (TAKARA, Japan) as previously described [12]. The first-strand complementary DNAs (cDNAs) were synthesized using a Prime Script RT reagent Kit (Perfect Real Time, TaKaRa) according to the manufacturer's instructions. The reverse transcription reaction was carried out at 37°C for 15 min and then inactivated at 85°C for 5 sec.

### Quantitative real-time polymerase chain reaction (qRT-PCR)

To assess the expression of miRNAs, SMO, Gli2 and TGF-β1 in U87-EGFRvIII cells, qRT-PCR was performed in triplicate in a CFX96 Real-Time System (Bio-Rad) using a FastStart Universal SYBR Green Master (ROX) (Roche Diagnostics) according to the manufacturer's instructions and expression levels were normalized to glyceraldehyde-3-phosphate dehydrogenase (GAPDH) and U6 as endogenous controls. To quantify gene expression, two-Step qRT-PCR was performed using hot start Taq at 95°C (15 s), with annealing and extension at 60°C (60 s) for 40 cycles, followed by a melting curve analysis. All qRT-PCR data were analysed using the 2^−ΔΔCt^ method [12]. The following gene-specific primers were used: miR-326 sense, 5′-CATCTG TCTGTTGGGCTGGA-3′; miR-326 anti-sense, 5′-AGG AAGGGCCCAGAGGCG-3′ (GenePharma); SMO sense, 5′-CTTCAGCTGCCACTTCTACGACTTC-3′; SMO anti- sense, 5′-TCGGGCGATTCTTGATCTCAC-3′ (Sangon Biotech, China); Gli2 sense, 5′-TGTAAGCAGGAGG CTGAGGT-3′; Gli2 anti-sense, 5′-GCTCGTTGTTGATG TGATGC-3′ (Sangon Biotech); TGF-β1 sense, 5′-GGC CAGATCCTGTCCAAGC-3′; TGF-β1 anti-sense, 5′-GTG GGTTTCCACCATTAGCAC-3′ (Invitrogen); GAPDH sense, 5′-TGGACTCCACGACGTACTCAG-3′; GAPDH anti-sense, 5′-CGGGAAGCTTGTCATCAATGGAA-3′ (Invitrogen); U6 RT, 5′-TGGTGTCGTGGAGTCG-3′; U6 sense, 5′-CTCGCTTCGGCAGCACA-3′; U6 anti- sense, 5′-AACGCTTCACGAATTTGCGT-3′ (Gene Pharma). The following oligonucleotide sequences were designed and purchased from GenePharma: miR-326 mimic sense, 5′-CCUCUGGGCCCUUCCU CCAG-3′; anti-sense, 5′-GGAGGAAGGGCCCAGAGGU U-3′; miRNA scrambled sense, 5′-UUCUCCGAA-CGU GUCACGUTT-3′; anti-sense, 5′-ACGUGACACGUUC GGAGAATT-3′.

### Cell count kit-8 and flow cytometry

EGFRvIII-DCs were added to autologous T lymphocytes at ratios of 1:200, 1:100, 1:50, or 1:10, 1:5, 1:1, or 2:1, 1:1, 0.5:1; cell proliferation was quantified using a Cell Counting Kit-8 (Dojindo) according to the manufacturer's instructions. Each experiment was performed in triplicate and repeated independently at least twice. Annexin V labelling was used to identify apoptotic cells. Apoptosis was quantified using annexin V labelling after transfection for 48 h. Cells were resuspended in binding buffer. Then, 5 μl FITC annexin V and 5 μl propidium iodide (BD Pharmingen, USA) were added and incubated for 15 min. Stained cells were analysed by flow cytometry (FACSCanto II, BD Biosciences) [12]. To detect the DC, the following monoclonal antibodies (all from Biolegend, USA) were used for FACS analysis: mouse anti-human CD1a (FITC-conjugated, Clone: HI149) and mouse anti-human CD83 (PE-conjugated, Clone: HB15e). The expression of CD83 and CD1a molecules on the surface of DCs were evaluated by flow cytometry. FITC conjugated anti-human CD1a and PE conjugated anti-human CD83 antibodies were diluted 1: 20 in FCM buffer (Phosphate-buffered solution (PBS) + 1%BSA + 0.09%NaN_3_). One million cells in 100 μl staining volume of each of the diluted antibodies and kept on ice for about 30 min in dark. After that, cells were washed twice with PBS, resuspended in 500 μl FCM buffer and analyzed by flow cytometry (FACS Canto II, BD Biosciences).

### Western blot

Western blotting assays were used to detect the expression of SMO and Gli2. The primary antibody contained rabbit anti-SMO (1:1000 dilution; Abcam), rabbit anti-Gli2 (1:1000 dilution; ZSGB-BIO), and mouse anti-GAPDH (1:1000 dilution; ZSGB-BIO). Following incubation with horseradish peroxidase-labelled (HRP) secondary antibody (ZSGB-BIO), protein bands were detected using a Fujifilm Las-4000 [12]. The density of specific protein bands was quantified after normalization to the density of the GAPDH band in the same sample.

### Luciferase reporter assay

Wild-type luciferase reporter plasmids GV272-TGF-β1-3′-UTR and GV272-SMO-3′-UTR were created that contained putative miR-326 binding sites for TGF-β1 and the SMO 3′-UTR respectively, downstream of the luciferase open reading frame (Genechem, China). Next, we transiently expressed these constructs in U87-EGFRvIII cells in the presence or absence of miR-326 mimics using Lipofectamine 2000 reagent (Invitrogen) according to the manufacturer's protocol. Firefly luciferase plasmid and miR-326 mimics or Scramble-miR were co-transfected with CV045 *Renilla* luciferase plasmid (Genechem) for normalization in 96-well plates. Following a 48 h incubation, luciferase activity was measured using the Dual-Glo luciferase assay system (E2920, Promega, USA). Normalized luciferase activity was reported as the ratio of Firefly Luciferase activity- to-*Renilla* Luciferase activity.

### Statistical analysis

All statistical analyses were performed using SPSS Graduate Pack 19.0 statistical software (SPSS, Chicago, IL, USA). *P*-values of less than 0.05 were considered to be statistically significant. All data, including mean values, SE, and one-way ANOVA, were calculated and analysed with Prism GraphPad software.

## SUPPLEMENTARY MATERIALS FIGURES AND TABLES



## Abbreviations

EGFRvIII: type III variant of epidermal growth factor receptor
PEPvIII: EGFRvIII peptide sequence
DCs: Dendritic cells
GBM: Glioblastoma
Shh: sonic hedgehog
GLI: Glioma-associated oncogene homologue
SMO: smoothened
LNs: lymph nodes
IL-13Ra2: interleukin-13 receptor alpha 2
GSA: glioma-specific antigen
MDSCs: marrow-derived stem cells
MoDC: monocyte-derived dendritic cells
mDC: mature DC
CTL: cytotoxic T lymphocytes
PBS: Phosphate-buffered solution
IFN-γ: gamma-interferon
ELISA: Enzyme-linked immunosorbent assay
TGF-β1: Transforming growth factor-beta 1
