# Supplementary material for: Combination epidermal growth factor receptor variant III peptide-pulsed dendritic cell vaccine with miR-326 results in enhanced killing on EGFRvIII-positive cells
Source: Oncotarget. 2017 Feb 17;8(16):26256–68. doi: 10.18632/oncotarget.15445 (PMC5432254; doi:10.18632/oncotarget.15445)
Supplement: Supplementary file 1 [file oncotarget-08-26256-s001.pdf]

Combination epidermal growth factor receptor variant III peptide-pulsed dendritic cell vaccine with miR-326 results in enhanced killing on EGFRvIII-positive cells

Supplementary Materials

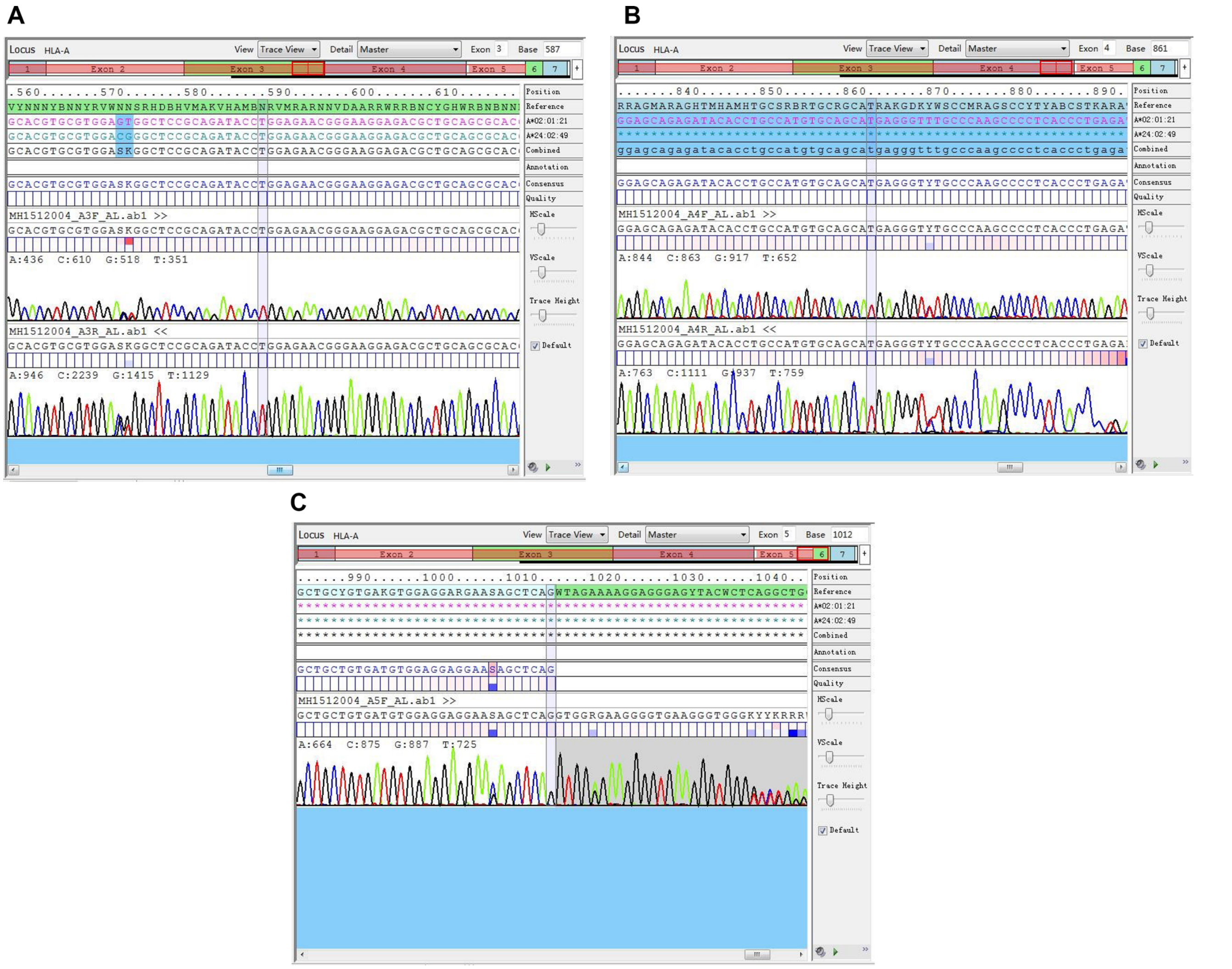

Supplementary Figure 1: The PCR products of HLA a2 positive PBMCs (Volunteer 1) were sequenced and analysis showed that they have the HLA a*0201 allele.

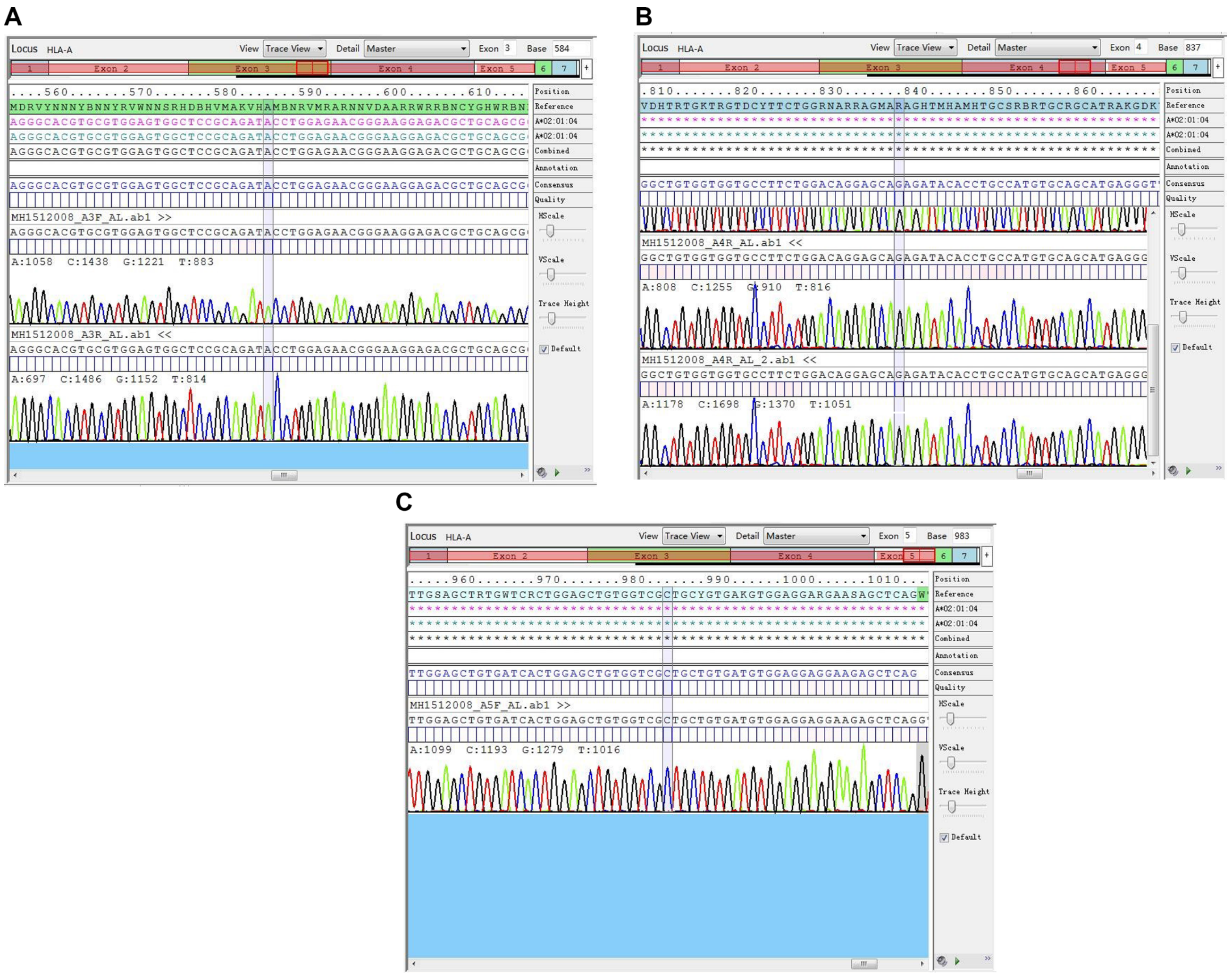

Supplementary Figure 2: The PCR products of HLA a2 positive PBMCs (Volunteer 2) were sequenced and analysis showed that they have the HLA a*0201 allele.

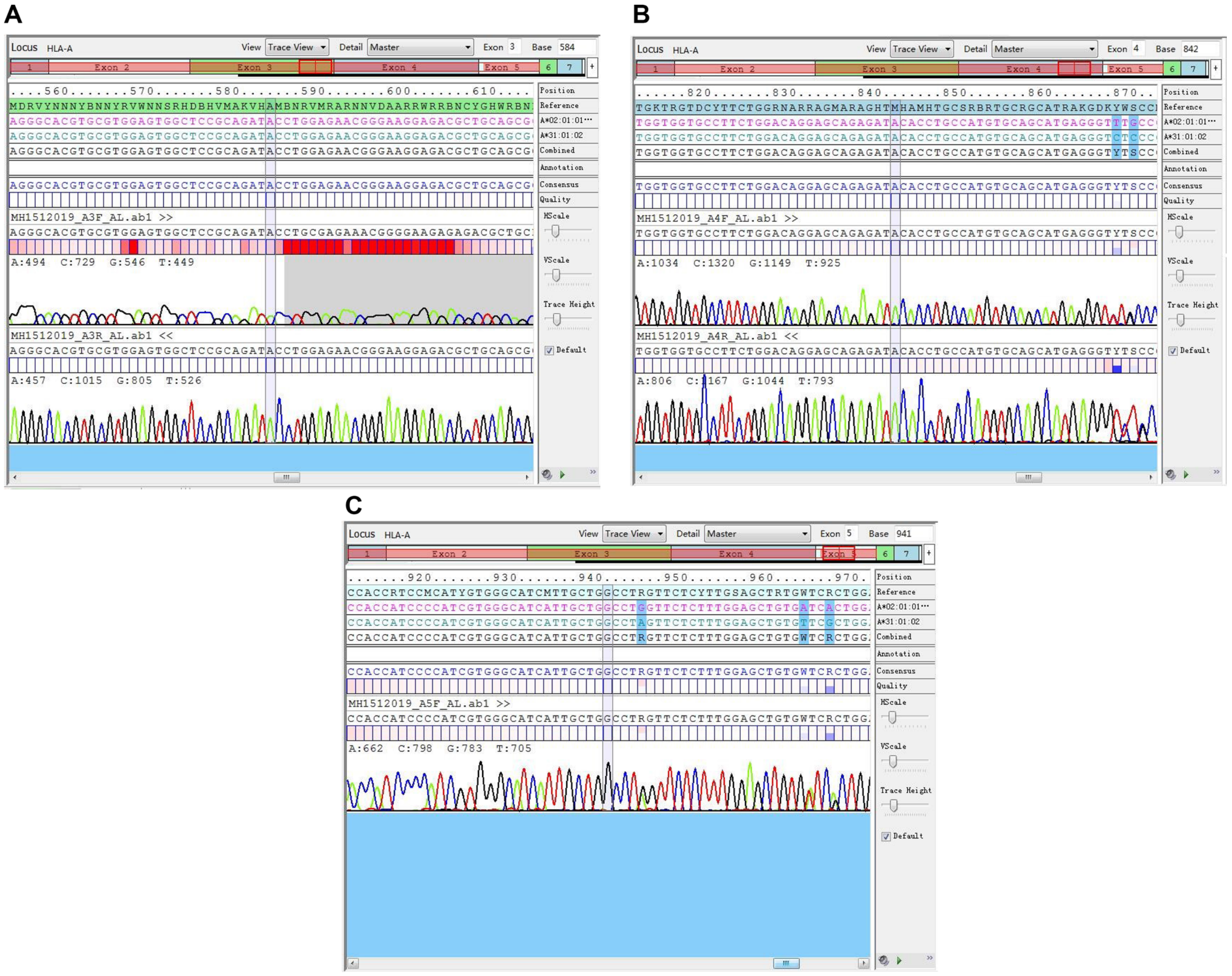

Supplementary Figure 3: The PCR products of HLA a2 positive PBMCs (Volunteer 3) were sequenced and analysis showed that they have the HLA a*0201 allele.

Supplementary Samples: The PCR-SSP process identified three volunteers' PBMCs that were HLA a2 positive.

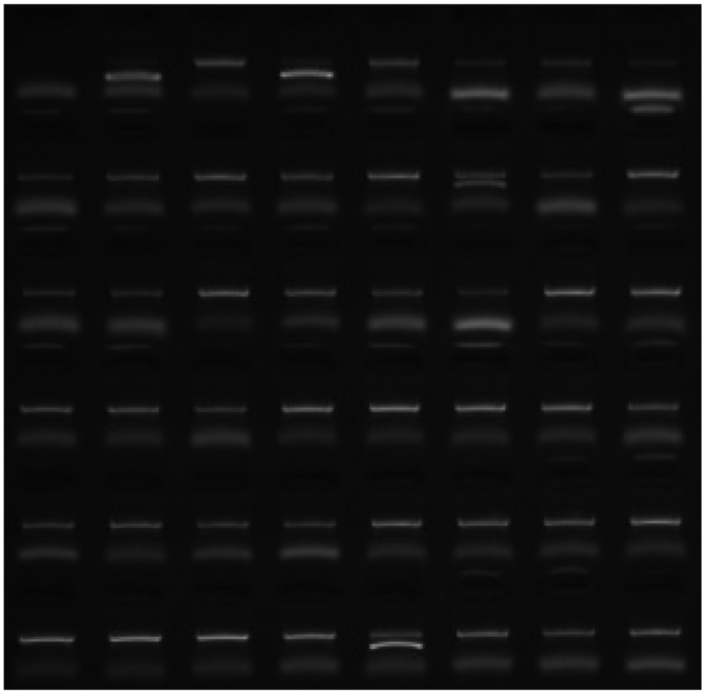

Supplementary Sample 1: A*02:01:01:01-01:01:04/01:03-01:30/01:32-01:60/01:62-01:69/01:71-01:81/01:84-01:104/01:106-01:108-01:115/11:01-11:07/16/20:01/22:01-24:02/29-35:03/40:01/40:02/42/43N/45/46/48/52/53N/55/59-60:02/64/66-71/74:01-76:01/77/80/82N/83N/85/88N-90/92-97:02/101:01/101:02/104/105/107/109/111/113:01N/113:02N/116/118-121/123/125N/128/129/131-134/138-141/146/147/150/151/153:01/153:02/157-162/164-168/173-177/181/182/184/185/187-189/192-207/211:01-218/220-225N/227N/228/231/233-235/237-241/247/249-252/254/256/257/260/262/263/266/269/270/273-277/283-285/287-289:02/291-294/296-299/301N/302/304-308/311-314N/316-318/321N/322/325-327/329/331/336/339-343/346-348/350N-352/354/356N/357/360-368/372/374/375/377-381/383-386/388-392/394-397/401/406/407/410/411/414/416/418/422-425/430/434/435/439N-443/445/446/448/455-462/464/467-469/479/481-483/485-488/490N/491/494/497-504/508-512/514N-516N/518-526/528/530/533-540N/542/547/548/551-555 || A*33:03:01-03:29/08-15/17/20/23-26/28-31/35-48/52/54-58/60/62/63/65/66/70-88/90/93-95. Positive Wells : 002,004,014,045.

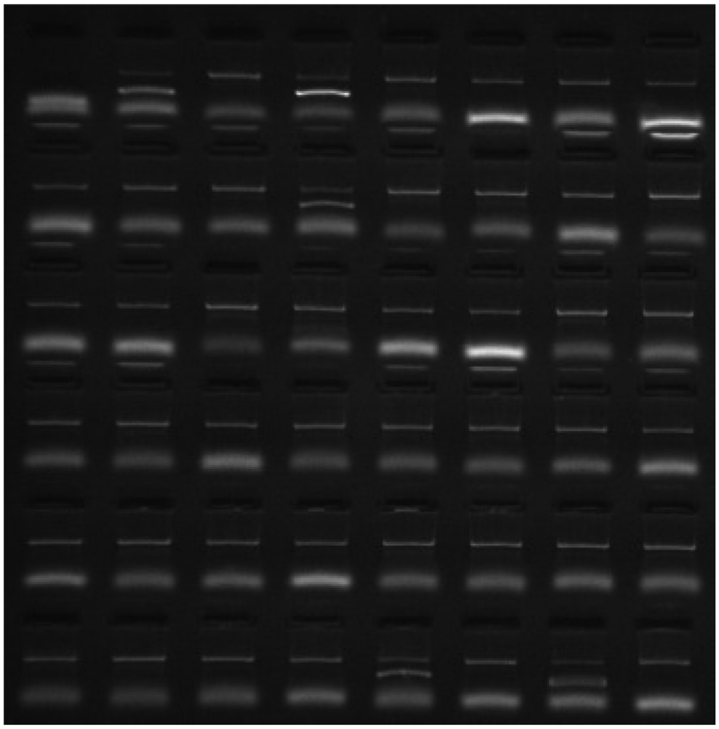

Supplementary Sample 2: A*02:01:01:01-01:01:04/01:03-01:30/01:32-01:60/01:62-01:69/01:71-01:81/01:84-01:104/01:106-01:108-01:115/11:01-11:07/16/20:01/22:01-24:02/29-35:03/40:01/40:02/42/43N/45/46/48/52/53N/55/59-60:02/64/66-71/74:01-76:01/77/80/82N/83N/85/88N-90/92-97:02/101:01/101:02/104/105/107/109/111/113:01N/113:02N/116/118-121/123/125N/128/129/131-134/138-141/146/147/150/151/153:01/153:02/157-162-/164-168/173-177/181/182/184/185/187-189/192-207/211:01-218/220-225N/227N/228/231/233-235/237-241/247/249-252/254/256/257/260/262/263/266/269/270/273-277/283-285/287-289:02/291-294/296-299/301N/302/304-308/311-314N/316-318/321N/322/325-327/329/331/336/339-343/346-348/350N-352/354/356N/357/360-368/372/374/375/377-381/383-386/388-392/394-397/401/406/407/410/411/414/416-/418/422-425/430/434/435/439N-443/445/446/448/455-462/464/467-469/479/481-483/485-488/490N/491/494/497-504/508-512/514N-516N/518-526/528/530/533-540N/542/547/548/551-555 || A*31:01:02:01-02/05/07-23/26-28/30-61/63-66/69-87/89,A*33:21/53. Positive Wells : 002,004,012,045.

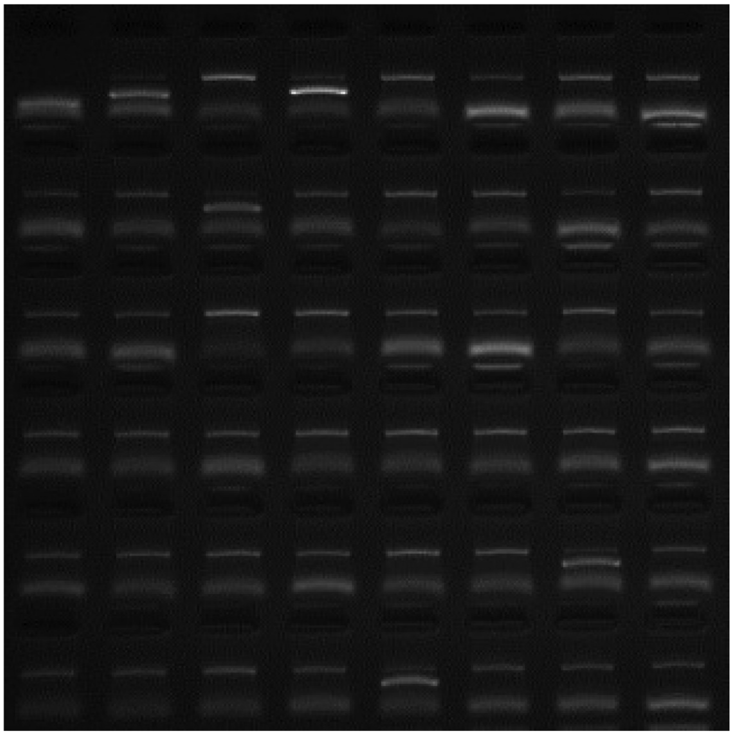

Supplementary Sample 3: A*02:01:01:01-01:01:04/01:03-01:30/01:32-01:60/01:62-01:69/01:71-01:81/01:84-01:104/01:106-01:108-01:115/11:01-11:07/16/20:01/22:01-24:02/29-35:03/40:01/40:02/42/43N/45/46/48/52/53N/55/59-60:02/64/66-71/74:01-76:01/77/80/82N/83N/85/88N-90/92-97:02/101:01/101:02/104/105/107/109/111/113:01N/113:02N/116/118-121/123/125N/128/129/131-134/138-141/146/147/150/151/153:01/153:02/157-162/164-168/173-177/181/182/184/185/187-189/192-207/211:01-218/220-225N/227N/228/231/233-235/237-241/247/249-252/254/256/257/260/262/263/266/269/270/273-277/283-285/287-289:02/291-294/296-299/301N/302/304-308/311-314N/316-318/321N/322/325-327/329/331/336/339-343/346-348/350N-352/354/356N/357/360-368/372/374/375/377-381/383-386/388-392/394-397/401/406/407/410/411/414/416/418/422-425/430/434/435/439N-443/445/446/448/455-462/464/467-469/479/481-483/485-488/490N/491/494/497-504/508-512/514N-516N/518-526/528/530/533-540N/542/547/548/551-555 || A*30:01:01-01:07/01:09/01:10/11:01/11:02/14L-17/19/20/23-25/30/31/35-43/48/49/52-54/56/58-60/62/63/65/71-73N/75/78N/81-83/86-89/91-93. Positive Wells : 002,004,011,039,045.
